# A beta-tubulin-based qPCR assay for monitoring of *Peronospora variabilis* and disease progression in quinoa

**DOI:** 10.3389/fpls.2026.1872093

**Published:** 2026-09-30

**Authors:** Valeria D. Palma-Encinas, Jorge F. Yaniquez Vedia, Micaela Claure-Mollo, Keveen F. Vasquez-Vargas, Paola Nogales-Ascarrunz, Susana Huanca, Patricia A. Mollinedo, Claes von Wachenfeldt, Allan G. Rasmusson

**Affiliations:** 1 Department of Biology, Lund University, Lund, Sweden; 2 Instituto de Investigaciones Químicas IIQ, Universidad Mayor de San Andrés UMSA, La Paz, Bolivia

**Keywords:** *Chenopodium quinoa*, chlorophyll determination, imaging, *Peronospora variabilis*, quantitative PCR

## Abstract

*Peronospora variabilis*, the causal agent of downy mildew disease, is an important pathogen affecting quinoa production worldwide. Conventionally its detection relies primarily on visual scoring of chlorosis, at times complemented by PCR-based assays targeting the cytochrome oxidase subunit 2 (*cox2*) or internal transcribed spacer (*ITS*) regions. In this study, we evaluated three candidate qPCR markers (*cox2*), NADH dehydrogenase subunit 1 (*nad1*), and β-tubulin (*btub*) and developed a *btub*-based qPCR assay for quantifying *P. variabilis* during infection. Among the three markers, the *btub* assay showed the best overall performance, with amplification efficiencies of 99–111% and coefficients of determination (R²) above 0.99 and was therefore selected for pathogen quantification. The *btub*-based qPCR assay enabled quantification of *P. variabilis* throughout disease progression and revealed three distinct phases of pathogen accumulation. Pathogen DNA accumulation was compared with chlorophyll, carotenoids, phenolics, betalains, and a rapid Red, Green, and Blue (RGB) colorimetric analysis. The pathogen accumulation curves revealed similar infection dynamics among cultivars previously classified as tolerant or susceptible, suggesting that differences in previously reported cultivar tolerance are not readily explained by pathogen accumulation in the sampled leaf tissue alone. This work introduces a qPCR assay targeting *btub* gene for quantifying *P. variabilis* in host plants, addressing the current incompleteness of validated molecular tools for tracking pathogen DNA accumulation during disease development and providing a valuable tool for linking pathogen growth with host physiological responses in future studies on plant–pathogen interactions.

## 1 Introduction

*Peronospora variabilis* is an oomycete responsible for downy mildew disease in Chenopodium crops such as quinoa (*Chenopodium quinoa*), cañahua (*C. pallidicaule)*, and white goosefoot (*C. album*) (Rollano-Peñaloza et al., 2021, 2022; Félix-Gastélum et al., 2022; Fondevilla et al., 2023). The disease is currently globally distributed across multiple regions where quinoa is cultivated. Dissemination of *P. variabilis* is postulated to occur primarily through the exchange of contaminated seeds (Testen et al., 2014). As quinoa cultivation expands globally, reports of downy mildew have become increasingly frequent (Testen et al., 2012; Choi et al., 2014; Rollano-Peñaloza et al., 2021). As an obligate biotroph, *P. variabilis* requires living quinoa hosts for propagation; this dependence poses significant methodological constraints for laboratory-based studies. Traditionally, downy mildew has been assessed by visual observation of symptoms, which in the case of *P. variabilis* include chlorosis, dwarfing, reduced yield, and, under conditions favorable for the pathogen, plant death. Symptom-based assessments are commonly complemented by molecular methods to enable accurate identification of *P. variabilis* (Danielsen and Munk, 2004; Colque-Little et al., 2021b).

Molecular identification of *P. variabilis* typically relies on amplification and sequencing of regions such as the internal transcribed spacer (*ITS*) of the ribosomal DNA and cytochrome oxidase subunit 2 (*cox2*) (Testen et al., 2014; Rollano-Peñaloza et al., 2022). Recently, *ITS*-based hydrolysis probe for qPCR was developed for pathogen detection in seeds and were also used for qualitative detection in foliar tissue (Testen et al., 2024). However, *ITS* is part of the multicopy rDNA array, and rDNA copy number can vary substantially within a species. In microbial eukaryotes, approximately two- to four-fold differences in rDNA copy number have been reported among fungal isolates, while copy number may also vary in response to physiological or environmental conditions and can change over generations (Lofgren et al., 2019; Lavrinienko et al., 2020; Wang et al., 2023). Such variation can complicate the relationship between *ITS* copy number and organism abundance and contribute to biases in rDNA-based quantitative analyses (Iwanaga et al., 2020; Lavrinienko et al., 2020). In contrast, single-copy loci may provide a more direct correspondence between target copy number and genome abundance and have therefore been proposed as alternative targets for quantitative molecular analyses (Lavrinienko et al., 2020).

The β-tubulin *(btub)* gene represents a promising candidate because *btub* has been reported as a single-copy gene in *Phytophthora cinnamomi* (Weerakoon et al., 1998). In addition, *btub* has been used as a reference gene for qPCR-based studies in other downy mildew pathogens, including *Peronospora belbahrii* (Cohen et al., 2017). Nevertheless, its suitability for DNA-based quantification of *P. variabilis* has not been investigated, and only a limited number of *btub* sequences are currently available in public databases. The mitochondrial genes *cox2* and the mitochondrial NADH dehydrogenase subunit 1 (*nad1*) also represent potential targets for pathogen quantification because they are widely used in oomycete phylogenetics and molecular diagnostics (Martin and Tooley, 2003; Robideau et al., 2011). However, the suitability of *btub*, *cox2*, and *nad1* for monitoring *P. variabilis* DNA accumulation during disease development has not been evaluated.

The primary symptom of *P. variabilis* infection is chlorophyll loss observed as chlorotic lesions in the quinoa leaves. In addition to these symptoms, grayish sporulation may develop on the abaxial leaf surface, constituting a direct visible sign of the pathogen. Microscopic examination of this growth reveals the sporangiophores and sporangia characteristic of the oomycete (Danielsen and Munk, 2004). Thus, the combined examination of disease symptoms and pathogen signs can provide valuable preliminary evidence of downy mildew disease and is widely used for evaluation in the field and for cultivar screening (Danielsen and Munk, 2004; Colque-Little et al., 2021b; Fondevilla et al., 2023). Chlorophyll loss, or the appearance of chlorotic lesions, generally expands from isolated interveinal spots to large yellowed areas as the pathogen colonizes different tissues. For this reason, chlorophyll degradation is commonly used as a proxy for disease progression; visual scoring of leaf yellowing is routinely applied to monitor disease progression under controlled and field conditions (Danielsen and Munk, 2004; Choi et al., 2010; Colque-Little et al., 2021a; Rollano-Peñaloza et al., 2022).

Chlorophyll content can be estimated using various methods, including non-destructive approaches suitable for field applications. The use of red, green, and blue (RGB) image analysis provides a simple and rapid approach for estimating chlorophyll content under field conditions (Sánchez-Sastre et al., 2020; Bodor-Pesti et al., 2025). Previous studies have estimated chlorophyll content in quinoa under nitrogen deficiency using RGB values, although this approach has not been applied to monitor disease progression (Riccardi et al., 2014). RGB-based phenotyping is not limited to nutrient stress and has also been used to assess rust disease progression in pea leaves (Osuna-Caballero et al., 2023). RGB-based chlorophyll estimation has also been applied in other plant species, such as *Arabidopsis thaliana* (Liang et al., 2017). Spectrophotometric quantification of other pigments, including carotenoids, phenolics, and betalains, is frequently used to evaluate plant stress responses because of their association with physiological stress (Sabir et al., 2025). However, these approaches are less commonly applied than chlorophyll-based assessments in studies of downy mildew disease.

Although molecular assays are available for *P. variabilis* detection, disease progression in quinoa is still assessed primarily through symptom phenotyping, and available molecular assays have not been validated for quantitative monitoring of pathogen DNA accumulation in planta during disease development. Moreover, the suitability of alternative nuclear and mitochondrial targets remains unexplored, and the potential of *btub, cox2*, and *nad1* for quantitative pathogen detection has not been evaluated. Therefore, there is a need for a sensitive and reliable assay that enables quantitative assessment of pathogen colonization in planta. This study aims to relate a single-gene PCR-based detection of *P. variabilis* with traditional symptoms of downy mildew disease, and to develop a qPCR assay for monitoring *P. variabilis* DNA accumulation in infected quinoa plants. Using this approach, we investigate the relationship between *P. variabilis* DNA accumulation and physiological indicators such as pigment content (chlorophyll, carotenoids, phenolics, and betalains). Furthermore, we evaluate chlorophyll extraction methods to assess their potential applicability in field or low-resource settings.

## 2 Materials and methods

### 2.1 *P. variabilis* isolation and maintenance

*P. variabilis* was collected from quinoa fields around the Salt Lake in Uyuni, Bolivia (19° 8′ 41.87″ S, 66° 46′ 15.16″ W). Infected quinoa plants were identified, and samples were collected as described previously (Rollano-Peñaloza et al., 2022)*. P. variabilis* was maintained on quinoa plants of the cultivar Real Maniqueña in a greenhouse at the Chemistry Department of UMSA University, La Paz, Bolivia.

To collect sporangia, infected leaves exhibiting visible sporulation were placed in 15 mL tubes containing 10 mL distilled water and gently agitated by inversion. After removing the leaves, the sporangial suspension was washed with distilled water, centrifuged twice at 2000 × g, and maintained at 4 °C until inoculation of new plants. The sporangia were quantified using a Neubauer chamber, following the method described by Rollano-Peñaloza et al. (2022) and adjusted to a density of 4 × 10^4^ sporangia/mL. Inoculations were performed using freshly collected sporangia, within approximately 1 h of collection. Sporangia were obtained from leaves exhibiting active sporulation.

### 2.2 *P. variabilis* identification and sequencing

Once *P. variabilis* infection was established in the greenhouse, symptomatic leaves were sampled for molecular identification. For DNA extraction, 50 mg of leaf tissue was ground to a fine powder in liquid nitrogen using a mortar and pestle and transferred to a microcentrifuge tube. DNA was extracted following the manufacturer’s instructions for the PureLink Genomic DNA Kit (Thermo Scientific, Carlsbad, CA, USA).

Polymerase chain reaction (PCR) amplification was performed using Taq DNA Polymerase with ThermoPol^®^ Buffer (M0267, New England Biolabs). Target regions included *cox2* (Testen et al., 2014), the *btub* gene (Göker et al., 2007), and the *nad1* gene (evaluated in this work). Primer sequences are listed in **Supplementary Table 1**. PCR conditions for amplification of *cox2* and *nad1* fragments were as follows: initial denaturation at 94 °C for 30 s; 30 cycles of 94 °C for 30 s, 50 °C for 30 s, and 68 °C for 1 min; followed by a final extension at 68 °C for 5 min. For the *btub* fragment, conditions were as follows: initial denaturation at 94 °C for 30 s; 30 cycles of 94 °C for 30 s, 55 °C for 30 s, and 68 °C for 1 min; followed by a final extension at 68 °C for 5 min. PCR products were subjected to 0.8% (w/v) agarose gel electrophoresis to confirm the presence of amplicons and the expected size. Five microliters of PCR products were purified using ExoSAP-IT™ Express PCR Product Cleanup (Thermo Scientific, CA, USA). PCR products were sequenced in both forward and reverse directions using the Sanger method (Eurofins, Germany). The GenBank accession numbers for *cox2*, *btub* and *nad1* are PZ698328, PZ698329 and PZ698330, respectively.

### 2.3 Plant material

Quinoa seeds were supplied by PROINPA (Quipaquipani, La Paz, Bolivia). Two quinoa cultivars were selected, Kurmi, known to be more tolerant, and Pandela, which has been used previously for maintaining *P. variabilis* diseases (Danielsen and Munk, 2004; Rollano-Peñaloza et al., 2022).

Two independent greenhouse experiments were conducted in Lund (Sweden) and La Paz (Bolivia). In Lund, plants were cultivated under controlled greenhouse conditions with a mean temperature of 19 °C (13–20 °C diurnal range) and a 12 h light/12 h dark photoperiod. Illumination consisted of natural sunlight supplemented with lamps (Osram, 400W, Sweden) equipped with bulbs (Philips MASTER GreenPower SON-T PIA, 400W, Belgium) and LED lamps (90W, input voltage 85–265 V, 50/60 Hz; manufacturer not specified), providing an average PPFD of 250 μmol m^-^² s^-^¹ (range: 196–289 μmol m^-^² s^-^¹).

In La Paz, plants were cultivated in a greenhouse under natural sunlight without supplemental lighting. The natural photoperiod during the experimental period was 11.2–11.8 hours, and the greenhouse temperature was controlled between 10 and 25 °C.

### 2.4 Experimental setup and *P. variabilis* inoculation

A compact cultivation system was employed at both experimental locations, Lund and La Paz, consisting of polypropylene tubes (4.5 cm diameter × 15 cm height) arranged together in a tray. Each tube was filled with standard greenhouse soil. Between three and five seeds were sown at a depth of 0.5 cm in the center of each tube and covered with a thin layer of sand sufficient to cover the seeds. After one week, seedlings were thinned to one per tube to ensure uniform growth.

Plants were grown until they developed three fully expanded pairs of leaves before inoculation. The trays with the plants were placed inside plastic containers and inoculated by spraying a suspension of *P. variabilis* sporangia (4 × 10^4^ sporangia/mL) or distilled water (control) onto the foliage until drip-off. Relative humidity inside the containers was monitored using a digital hygrometer. During the first 48 h after inoculation, relative humidity was maintained at 90–100% to promote sporangia germination. Thereafter, the lids were partially opened, resulting in relative humidity values of approximately 20–60% during the remainder of the experiment (**Supplementary Table 2**). To prevent accidental spread of the pathogen to control plants after the inoculations, the containers were placed in two identically equipped greenhouse chambers.

In Lund, cultivar Kurmi was used, with three biological replicates per treatment and sampling day. The experiment conducted in Lund was designed to develop and validate a qPCR assay. For each plant, one leaf from the third pair of leaves was collected for analysis. Each leaf represented one biological replicate. Based on the biological variability observed during this experiment, the number of biological replicates was increased in a subsequent experiment performed. In La Paz, eight biological replicates were included per cultivar, treatment, and sampling day. For each plant, three leaves were collected (one from each of the three pairs of opposite leaves), measured with a colorimeter, and scanned for image-based assessment following the protocol described in Section 2.7. The leaves were then pooled, flash-frozen in liquid nitrogen, and homogenized while frozen. Subsamples of the homogenized material were allocated for downstream analyses. Five biological replicates were randomly selected for qPCR analysis, whereas eight biological replicates were used for spectrophotometric analysis. Material for spectrophotometric measurements was lyophilized before extraction.

### 2.5 Primer design and quantification of *P. variabilis* with qPCR

Three genes were evaluated for quantifying *P. variabilis*: *btub*, *cox2*, and *nad1*. Short target regions were selected from larger sequences previously obtained (**Supplementary Table 1**) using Primer3Plus software (Untergasser et al., 2012). Primer sequences and expected amplicon sizes are listed in **Supplementary Table 3**.

Quantitative PCR (qPCR) was performed using Maxima SYBR Green qPCR Master Mix (2×) (Thermo Fisher Scientific Baltics, Vilnius, Lithuania) on a Bio-Rad CFX384 Real-Time PCR System. Reactions were carried out in a 5 μL final volume containing 0.4 μM of each primer and 2 μL of template DNA. The thermal cycling program consisted of: 1 cycle at 50 °C for 2 min, 1 cycle at 95 °C for 10 min, 35 cycles of denaturation at 95 °C for 15 s, annealing at 63 °C for 30 s, and extension at 72 °C for 30 s, followed by a melting curve analysis for 30 min.

### 2.6 Specificity and efficiency assay

The performance of qPCR assay in the presence of host DNA was evaluated by mixing *P. variabilis* DNA extracted from isolated sporangia (obtained from 0.5 cm leaf disks) with DNA from healthy quinoa leaf disks. Approximately 20 ng of quinoa DNA (obtained from healthy 0.5 cm leaf disks) was mixed with four successive 10-fold serial dilutions of *P. variabilis* DNA prepared with the highest amounts of 20 or 50 ng of DNA per qPCR reaction. The amount of quinoa DNA (20 ng) was kept constant across all dilutions. A negative control containing quinoa DNA without *P. variabilis* DNA was included.

Primer efficiency was determined using two independent standard curves generated from the same dilution series described above, one for the Lund experiment and one for the La Paz experiment. Each standard curve was used to quantify samples from its corresponding experiment. Each dilution was analyzed in technical triplicate using the qPCR conditions described above. The lowest concentration consistently amplified across technical replicates and remaining within the linear range of the standard curve was considered the lower limit of reliable quantification. CT values were plotted against the logarithm of the amount of *P. variabilis* DNA per reaction, and amplification efficiency (*E*) was calculated from the slope of the standard curve using the formula:


$$E=(10^{\;-\frac{1}{slope}}-1)\;x\;100$$

Both quinoa and *P. variabilis* DNA were extracted using the PureLink Genomic DNA Kit (Thermo Scientific, Carlsbad, CA, USA). Amplification products were assessed by agarose gel electrophoresis and melting curve analysis to assess the presence of non-specific amplification.

To evaluate qPCR performance in infected leaf tissue, samples were prepared from infected leaves, selecting areas with varying degrees of sporulation. Disks were excised using a sterilized metal punch (0.5 cm internal diameter). All samples were immediately flash-frozen in liquid nitrogen, homogenized for 1 min using a TissueLyser (Qiagen), and genomic DNA was extracted using the PureLink Genomic DNA Kit (Thermo Scientific, Carlsbad, CA, USA).

For subsequent analyses of experimentally infected leaf samples from the Lund and La Paz experiments, total DNA was extracted using the same PureLink Genomic DNA Kit (Thermo Scientific, Carlsbad, CA, USA) described above. Extracted DNA concentration was adjusted to 10 ng/μL prior to qPCR analysis. Two microliters of template DNA were used per reaction, corresponding to 20 ng of total DNA per reaction.

### 2.7 Chlorophyll estimation in infected plants

Physiological and pigment analyses were performed on samples from the La Paz experiment using eight biological replicates per cultivar, treatment, and sampling day (n = 8), with each individual plant considered a biological replicate. Two approaches were used to estimate chlorophyll content in quinoa leaves: RGB analysis and spectrophotometry. For the RGB analysis, chlorophyll content was estimated using RGB values obtained from a colorimeter (ColorMuse, Variable, USA) and from scanned images. The colorimeter was calibrated according to the manufacturer’s instructions, and measurements were taken at four positions per leaf in a cross pattern. The average of these readings was considered for analysis.

Images were captured using a Canon image RUNNER 153 ADVANCE DX C5860i scanner at 600 × 600 dots per inch resolution and analyzed using FIJI (ImageJ; Schindelin et al., 2012). The background and petiole up to the leaf base were removed before analysis from each image. Individual leaves were analyzed using a custom macro in FIJI. RGB values were extracted by splitting each image into red, green, and blue channels using the “RGB Stack” function. To avoid extreme pixel values (pure black or white), a threshold range of 10–240 was applied to all channels using the “Default dark no-reset” setting. The macro then executed the “Measure Stack…” function to obtain intensity values for each channel within the defined threshold.

The resulting RGB values from the colorimeter and FIJI analysis were used to estimate chlorophyll content by applying the equation described by Riccardi et al. (2014):


$$Chlorophyll=\;exp{\;}^{\left(-\;0.0102\text{R }-\;0.0287\text{G }+\;0.0071\text{B }+5.5337\right)}$$

R: red channel intensity; G: green channel intensity; B: blue channel intensity.

#### 2.7.1 Spectrophotometric chlorophyll and carotenoid quantification

Chlorophyll and carotenoid concentrations were determined following the methodology of Lichtenthaler (1987) with minor modifications. Briefly, 10 mg of lyophilized leaf tissue were weighed and placed in 15 mL tubes. Three milliliters of methanol were added, and samples were incubated at room temperature for 40 h under continuous orbital shaking.

Absorbance readings were performed in triplicate using a 96-well plate format at wavelengths of 652 nm, 665 nm, and 470 nm. The absorbance values were used to calculate chlorophyll a, chlorophyll b, total chlorophyll, and total carotenoid concentrations according to Lichtenthaler’s equations.

#### 2.7.2 Quantification of total phenolic compounds

Total phenolic compounds (PCs) were quantified using the Folin–Ciocalteu method as described by Peñarrieta et al. (2008), with minor modifications. Results were expressed as gallic acid equivalents (GAE). A 1000 ppm gallic acid stock solution was prepared in 80% acetone and serially diluted to produce a calibration curve, with final concentrations of 10, 20, 30, 40, and 50 ppm.

For each sample, 30 µL of methanolic extract was mixed with 600 µL of 10% Folin–Ciocalteu reagent and incubated for 5 min. Subsequently, 300 µL of 7.5% sodium carbonate was added, and the mixture was stirred and incubated at 45 °C for 30 min. Absorbance was measured in triplicate at 765 nm using a spectrophotometer.

#### 2.7.3 Quantification of betalains

Betalains were extracted from 50 mg of tissue with 500 µL of 50% methanol solution containing ascorbic acid (pH 4). Samples were incubated for 30 min at 4 °C. This extraction process was repeated a total of 3 times. The combined extract from each sample was transferred into three wells of a 96-well plate. Absorbance was measured using a UV–VIS spectrophotometer (BioTek Instruments, Winooski, VT, USA) at 538 nm for betacyanins and 483 nm for betaxanthins. Betalain concentrations were subsequently calculated using the equations described by Tabio-García et al. (2021).

#### 2.7.4 Thin-layer chromatography

Pigment separation was carried out on fresh and freeze-dried leaves. Extracts were obtained using methanol P.A. grade (BIOPACK, Buenos Aires, Argentina) at room temperature, following the methanol extraction procedure described above. Fresh and frozen leaves were included as a control. The extraction of control samples was performed using methanol and MgSO_4_ P.A. grade (Merck, Darmstadt, Germany) as described by Quach et al. (2004). Thin-layer chromatography was performed on Silica gel 60 F254 plates (Merck, Darmstadt, Germany). For each sample, 10 μL of methanolic extract was applied to the TLC plate. Plates were placed in a pre-saturated TLC chamber containing the 5-solvent system described by Quach et al. (2004). After development, plates were removed, air-dried, and examined under visible and UV light to assess pigment separation profiles (Wagner et al., 1984).

### 2.8 Statistical analysis

For the La Paz experiment, temporal differences in pathogen DNA accumulation and physiological variables, including chlorophylls, carotenoids, phenolic compounds, and betalains, were evaluated using one-way analysis of variance (ANOVA), with sampling day as the factor. Analyses were performed separately for each cultivar and treatment (*P. variabilis*-inoculated and control plants). Differences in pathogen DNA accumulation between cultivars were evaluated separately at each sampling day using one-way ANOVA. When significant differences among sampling days were detected, means were compared using Tukey’s honestly significant difference (HSD) *post hoc* test. Statistical significance was set at P < 0.05.

Linear regression analyses were used to evaluate the relationship between spectrophotometrically determined chlorophyll content and chlorophyll estimates obtained from RGB measurements. Pearson correlation coefficients were calculated to assess associations among pathogen DNA accumulation and the physiological variables evaluated. All analyses and plots were performed using R (version 4.4.1).

## 3 Results

### 3.1 *P. variabilis*-induced foliar damage

To evaluate disease progression under greenhouse conditions, quinoa plants with at least three fully developed leaves were inoculated with a *P. variabilis* suspension or with water as a control. After inoculation, plants were maintained under controlled growth conditions and photographed daily for eight days post-inoculation (dpi) to document visual changes in whole plants and detached leaves. Monitoring continued until the inoculated leaves were lost (**Figure 1**).

**Figure 1:**
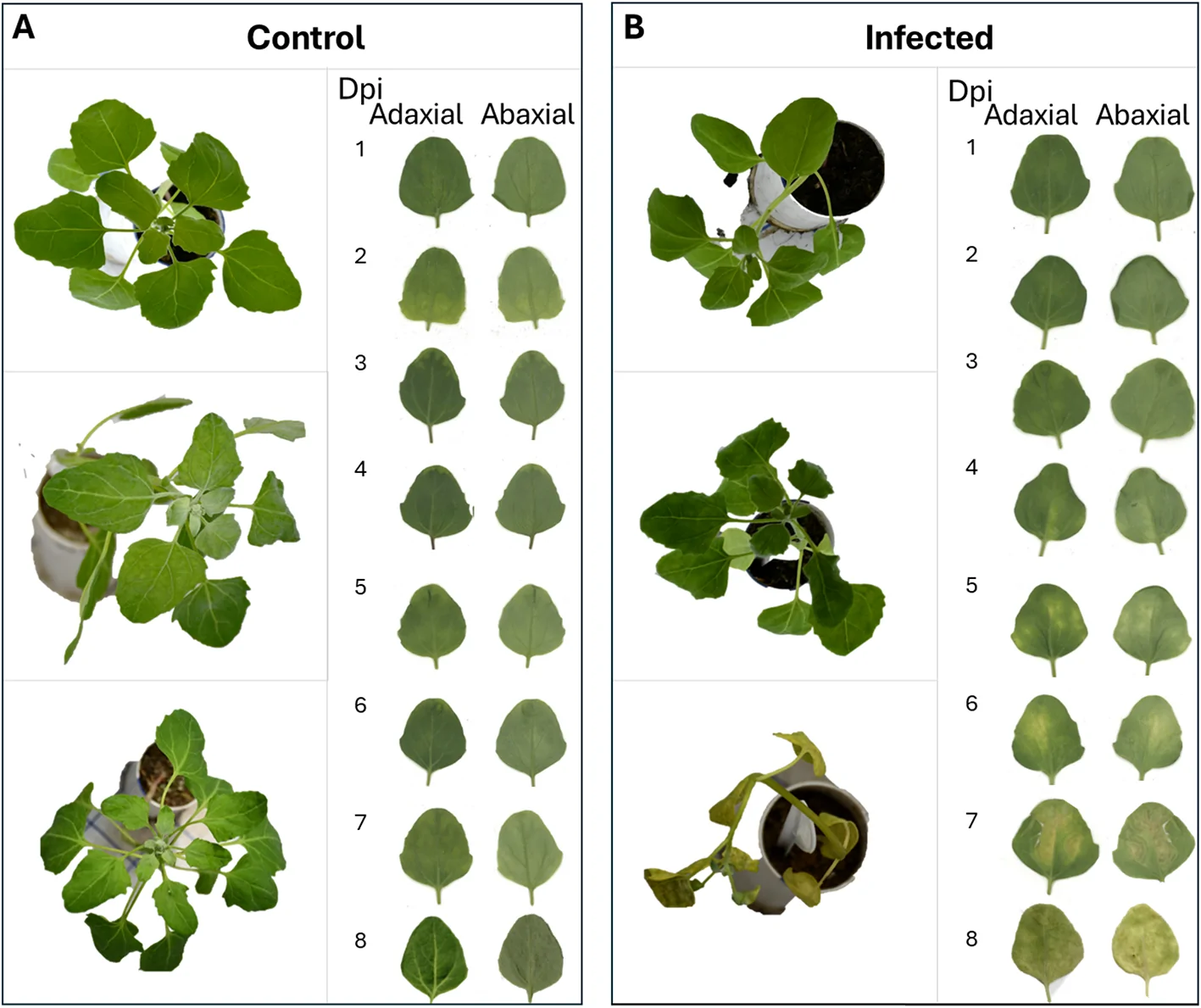
Infection symptoms in quinoa, cv. Kurmi. Disease symptoms were evaluated on healthy and infected Kurmi variety quinoa plants at 7 weeks of age. **(A)** Plants and the second pair of leaves foliar-inoculated with water (control), monitored over 8 days **(B)**. Plants and the second pair of leaves foliar-inoculated with *P. variabilis* (infected), monitored for 8 dpi.

High humidity (96-100%) was maintained during the first 48 h after inoculation, followed by 20-60% humidity for the remaining period. Chlorotic symptoms, either general or localized, appeared between 4 and 5 dpi. From this point onward, a clear difference between infected and control plants was observed; infected plants drastically lost chlorophyll and plant vigor (**Figure 1**). *P. variabilis* spores could be observed during this period as well, but they were more abundant on days 6 and 7. Spore development on the leaves triggered the onset of senescence. Sampling concluded at 8 dpi when leaves began to abscise.

### 3.2 Efficiency and specificity of *Peronospora variabilis* qPCR assay

DNA was extracted from infected quinoa leaves, and PCR amplification was performed using primers targeting *cox2*, *nad1*, and *btub* genes (**Supplementary Table 1**), followed by Sanger sequencing. The *cox2* gene is commonly used for *P. variabilis* identification, and numerous sequences are available in NCBI GenBank. We used the *cox2* fragment for species confirmation. PCR amplification yielded an expected 610 bp product for *cox2*, and BLAST analysis showed 100% similarity with *P. variabilis* isolates deposited in GenBank. In contrast, *btub* and *nad1* genes are less frequently employed for identification purposes. Their sequences were used for the design of qPCR primers. All oligonucleotide sequences utilized in this study are provided in **Supplementary Table 1**.

#### 3.2.1 Assay specificity and efficiency of *btub*, *cox2* and *nad1* genes for *P. variabilis* in the host-pathogen system

After sequencing, qPCR primers were designed; primer sequences and amplicon sizes are provided in **Supplementary Table 3**. DNA was extracted from the sporangial suspension, and assay efficiency and amplification performance were evaluated using a dilution series (**Figure 2A**). To assess the effect of the host DNA on assay performance, 20 ng of quinoa genomic DNA was mixed with two dilution series of *P. variabilis* DNA, with the highest amount corresponding to 20 or 50 ng. Two standard curves were obtained, with coefficients of determination of R² = 0.9992 and 0.9923 (**Figure 2A**). The measured amplification efficiencies were 99% and 111%, respectively. The assay showed a linear quantification range down to 0.002 ng of *P. variabilis* DNA per reaction. Amounts below this level produced inconsistent amplification and were therefore considered outside the reliable quantification range.

**Figure 2:**
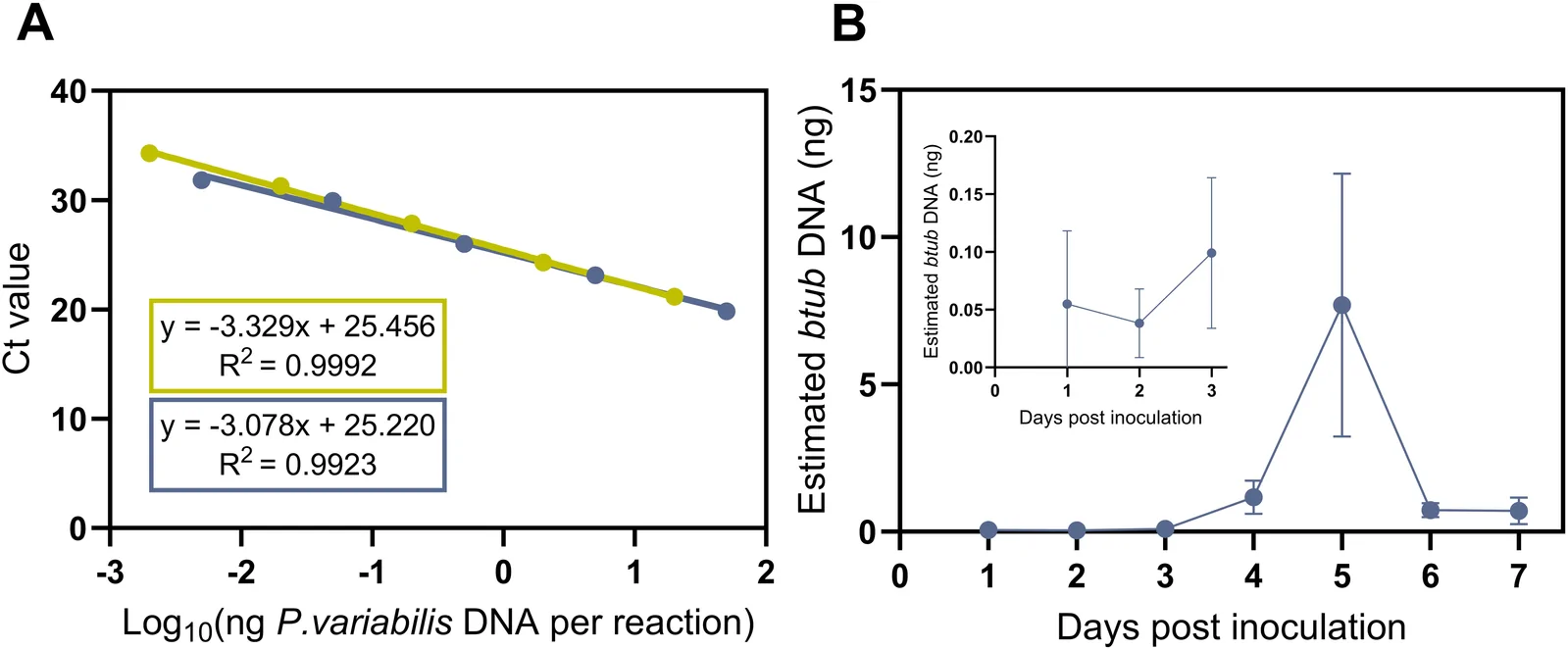
*P. variabilis* DNA amplification by qPCR targeting the *btub* gene. **(A)** Standard curve generated using 10-fold serial dilutions of *P. variabilis* genomic DNA. The experiment was performed twice, starting from 20 ng (green line) and 50 ng (blue line) of *P. variabilis* DNA per qPCR reaction, each mixed with 20 ng of quinoa genomic DNA. **(B)** Propagation curve of *P. variabilis* on quinoa cultivar Kurmi based on quantitative real-time PCR amplification of the *btub* gene. The curve illustrates the progression of infection over 7 days post inoculation. Data represent mean ± standard error (n = 3 per day).

Healthy quinoa genomic DNA and no-template control (water) were included as negative controls and did not produce detectable amplification within 35 cycles for the *btub* assay. In contrast, agarose gel electrophoresis demonstrated that the nad1 and COX2 primer sets were prone to dimer formation and exhibited amplified non-target quinoa DNA. In addition, both assays showed reduced linearity, particularly at the lowest DNA concentrations, with coefficients of determination (R²) of 0.9507 (*cox2*) and 0.9538 (*nad1*) and amplification efficiencies of 77% and 67%, respectively (**Supplementary Figure 1**). Due to these limitations, primers targeting *cox2* and *nad1* genes were therefore excluded, and only the *btub* marker was selected for downstream quantification.

Using btub primers and the standard curve (**Figure 2A**), we quantified *P. variabilis* proliferation in infected plants. Over the seven-day infection period, quinoa plants (cv. Kurmi) displayed three distinct phases of pathogen accumulation (**Figure 2B**). The first two days corresponded to a lag phase with minimal change in *P. variabilis* DNA levels. A pronounced increase occurred between days 3 and 5, culminating in the highest pathogen load on day 5. From days 6 to 7, levels decreased but remained substantially above those detected during the initial lag phase.

#### 3.2.2 Leaf tissue does not interfere with *P. variabilis* DNA quantification, but spores compromise amplification efficiency

We then decided to continue evaluating *btub* gene as a target for qPCR using leaves sampled 7 dpi. Natural infection samples represent a complex matrix of quinoa tissue and various *P. variabilis* structures, including hyphae, sporangia, and spores. To evaluate the contribution of *P. variabilis* sporangia we took discs of 0.5 cm from an infected leaf. As a reference, 10^4^ sporangia were also added on a disc from a healthy plant and processed as the other discs. As observed in **Figure 3** the *P. variabilis* levels correlate with visible sporulation. Although we could not determine if internal growth was uniform, all sampled discs contained higher amounts of *P. variabilis* than the reference sample, indicating that *P. variabilis* DNA from both hyphae and sporangia was detected.

**Figure 3:**
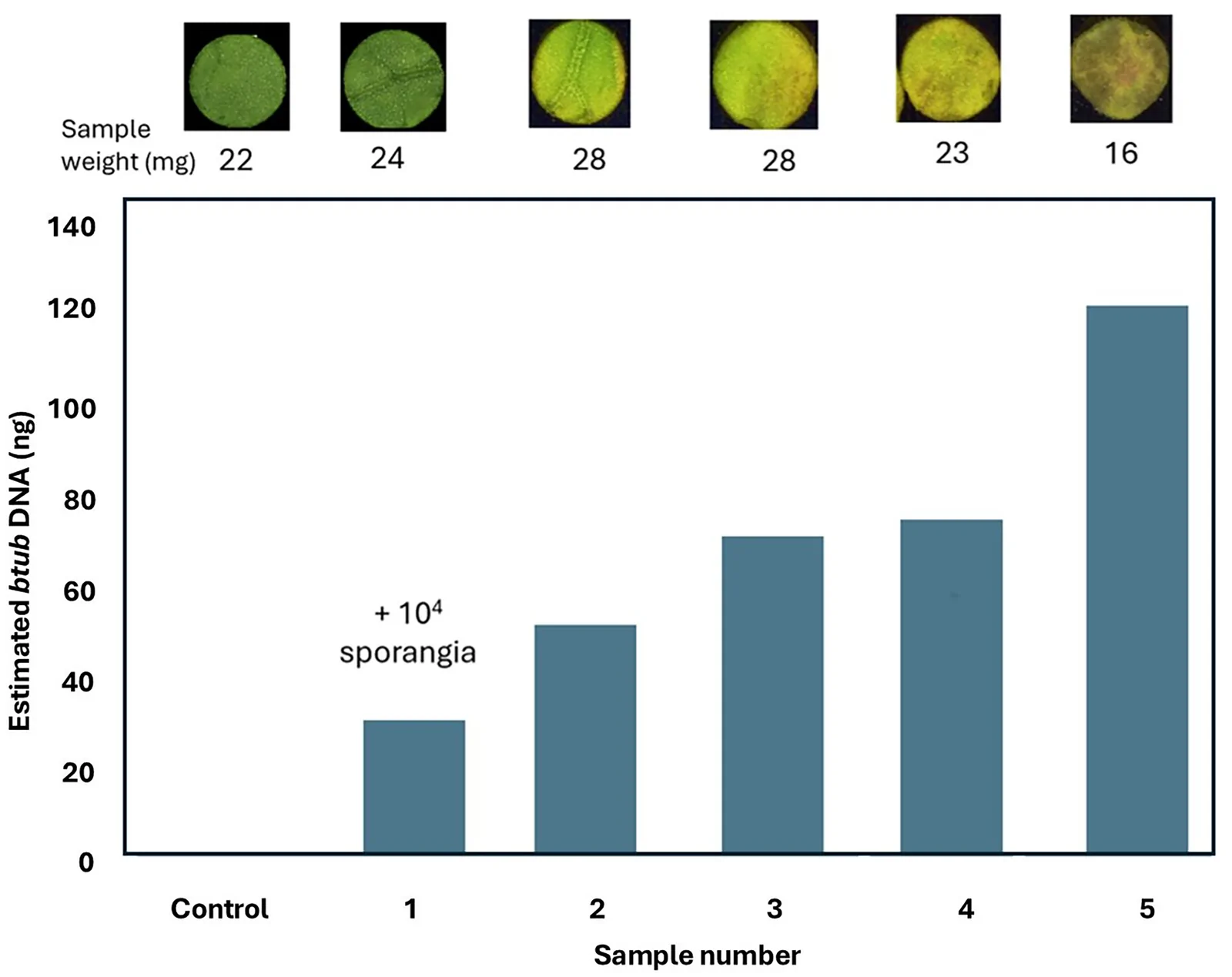
Estimated amount of *P. variabilis* DNA (ng) detected in different samples. The control sample corresponds to a quinoa leaf disc from healthy tissue. Sample 1 contains *P. variabilis* DNA extracted from 10^4^ sporangia mixed with 24 mg of healthy quinoa leaves. Samples 2–5 correspond to quinoa leaf discs collected 7 dpi from different visibly infected areas of the same leaf (n = 1), selected to represent visually increasing levels of sporulation, and analyzed separately. These samples were not independent biological replicates; therefore, no statistical comparison was performed. DNA quantity was calculated using the standard-curve equation in **Figure 2**.

### 3.3 Quantifying *P. variabilis* between different degrees of tolerance to the disease

The dynamics of *P. variabilis* propagation exhibited a clear temporal pattern, with a decrease in pathogen DNA between days 5 and 6 (**Figure 2B**). To evaluate whether pathogen quantification could be applied under more realistic conditions, a larger greenhouse experiment was conducted at the Universidad Mayor de San Andrés, La Paz (Bolivia), following the same inoculation and sampling procedures described above. In addition to pathogen quantification, several physiological parameters were measured, including chlorophyll, carotenoids, phenols, and betalains.

As shown in **Figure 4A**, P*. variabilis* DNA increased rapidly after day 4 in both cultivars, reaching maximum levels between days 6 and 7, then declined at day 8. This pattern was consistent with the three previously described phases (**Figure 2B**) of pathogen propagation and was observed in both Kurmi and Pandela. Likewise, chlorophyll content decreased progressively during infection in both cultivars (**Figure 4B**). No significant differences were detected between cultivars at any sampling day for either pathogen DNA accumulation or chlorophyll content, indicating similar temporal dynamics of infection and symptom development. *P. variabilis* DNA was not detected in control plants.

**Figure 4:**
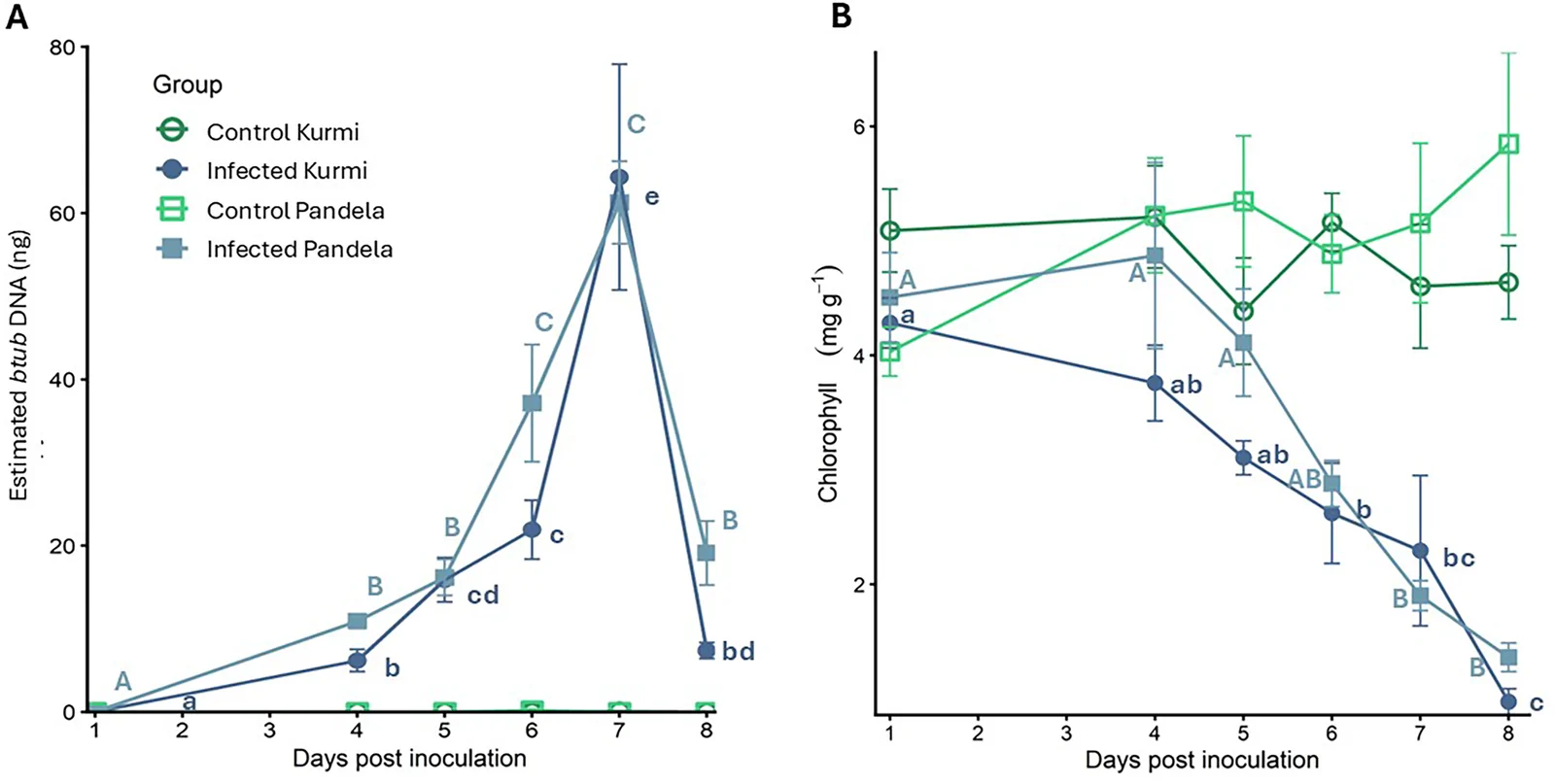
Pathogen quantification and chlorophyll content during *P. variabilis* infection in the quinoa cultivars Pandela and Kurmi. **(A)** Pathogen DNA accumulation was estimated by qPCR targeting the *β-tubulin* (*btub*) gene in Kurmi and Pandela following inoculation. **(B)** Total chlorophyll content (mg g^-^¹ DW) measured during infection. Data represents the mean ± standard error (n = 5 biological replicates per sampling day). Different lowercase letters (Kurmi) and uppercase letters (Pandela) indicate significant differences among sampling days within the infected treatment according to one-way ANOVA followed by Tukey’s Honestly Significant Difference (HSD) test (*P* < 0.05).

To further evaluate the relationship between chlorophyll content and pathogen progression, chlorophyll was quantified using both non-destructive RGB-based estimation and traditional spectrophotometric methods. Correlation between chlorophyll estimation methods ranged from R² = 0.61 to 0.80 (**Supplementary Figure 2**). Chlorophyll dynamics during *P. variabilis* infection were assessed in cultivars Kurmi and Pandela (**Figure 4B**). In both cultivars, chlorophyll content decreased in infected plants compared to water-inoculated controls. Pandela displayed slightly higher chlorophyll levels than Kurmi at early stages, but both cultivars showed pronounced decline from 7 and 8 days post-inoculation, while chlorophyll levels in controls remained stable (**Figure 4B**).

The estimated pathogen amount was compared with physiological parameters related to pigment and secondary metabolite accumulation (**Figure 5**). When expressed relative to total chlorophyll, phenolic compounds increased progressively during infection, with higher phenols/chlorophyll ratios observed in infected plants compared to controls, particularly at later time points, and more so in the Kurmi cultivar than in Pandela (**Figure 5**). A similar trend was observed for betalains normalized to chlorophyll, which showed similarly increased betalains/chlorophyll ratios in infected plants during disease progression in both cultivars (**Figure 5**).

**Figure 5:**
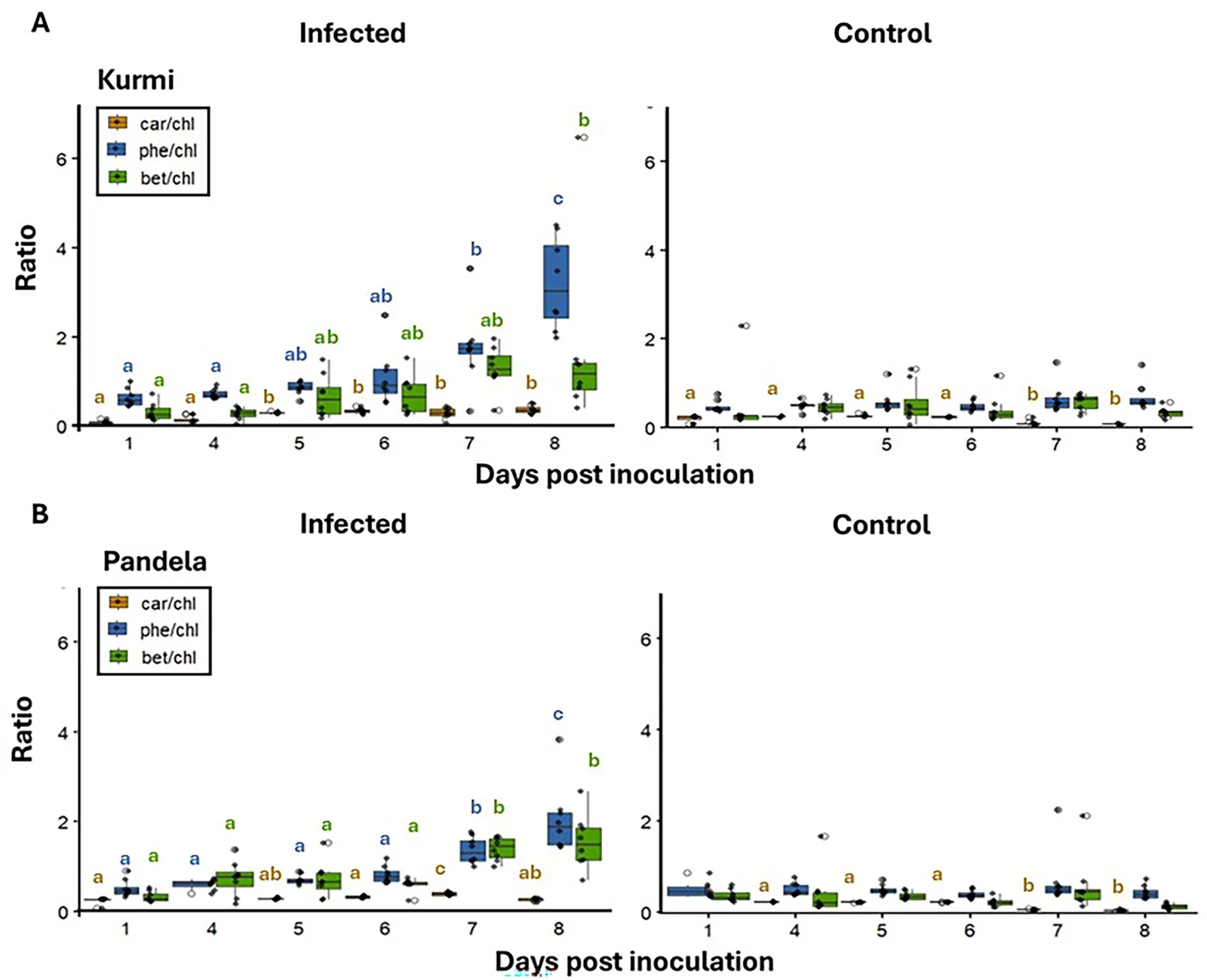
Physiological responses during *P. variabilis* infection in quinoa cultivars. Ratios of total phenolic compounds to total chlorophyll (phenols/chlorophyll), betalains to total chlorophyll (betalains/chlorophyll), and carotenoids to total chlorophyll (carotenoids/chlorophyll) were measured during disease progression. **(A)** Infected and control plants of the Kurmi cultivar. **(B)** Infected and control plants of the Pandela cultivar. Data represent n = 8 biological replicates per day. Different letters indicate significant differences among sampling days within each treatment, according to one-way ANOVA followed by Tukey’s HSD test (P < 0.05).

In contrast to chlorophyll, carotenoid content closely followed chlorophyll dynamics during the sampling days, but phenols and betalains did not (**Supplementary Figure 3**). Distinct temporal patterns were observed between cultivars: in Kurmi, carotenoid levels decreased at early time points following inoculation, whereas carotenoid content in Pandela remained relatively stable during the initial stages of infection (**Supplementary Figure 3**). A strong association between carotenoid and chlorophyll content was confirmed by thin-layer chromatography (TLC) analysis (**Supplementary Figure 4**). Based on this analysis, samples showing inconsistent carotenoid–chlorophyll profiles were excluded from further analysis. At later stages of the experiment, carotenoid content decreased after day 6 post-inoculation in both control and infected plants, mirroring the decline observed for total chlorophyll (**Supplementary Figure 5**).

## 4 Discussion

Chlorosis and leaf damage are widely used indicators of disease severity in quinoa, as these symptoms often precede more severe outcomes such as stunting, premature defoliation, and yield losses. The decline in chlorophyll content observed after *P. variabilis* inoculation in the quinoa cultivars Pandela and Kurmi is consistent with typical physiological responses associated with downy mildew infection and has been previously reported in quinoa (Rollano-Peñaloza et al., 2022; Danielsen and Munk, 2004; Colque-Little et al., 2021a; Fondevilla et al., 2023). Similar morphological responses have been reported across diverse plant–pathogen systems, where biotic stress leads to downregulation of photosynthesis-related parameters (Berger et al., 2007; Bilgin et al., 2010; Scholes and Rolfe, 2009). As a consequence, these parameters are commonly employed in screening programs to differentiate susceptible from tolerant cultivars. However, a purely chlorophyll content-based disease diagnosis can be confounded by factors unrelated to pathogen development. Colque-Little et al. (2021a) reported significant differences in disease severity and sporulation among quinoa accessions from distinct ecoregions, indicating that disease-associated traits may reflect genotype–environment interactions in addition to pathogen pressure (Poland et al., 2009; Corwin and Kliebenstein, 2017). Further, nitrogen fertilization in *Plantago ovata* can increase chlorophyll content and overall plant growth, although downy mildew severity is increased, likely due to increased nitrogen availability (Mandal et al., 2008).

Other disease-related parameters, such as sporulation timing and intensity, and plant survival following *P. variabilis* infection, are less frequently evaluated under greenhouse conditions. A comparable approach was reported by Rollano-Peñaloza et al. (2022), who inoculated quinoa plants (cv. Kurmi) with a *P. variabilis* suspension and monitored plant survival up to 21 dpi. In that study, the Kurmi cultivar developed chlorosis yet survived infection throughout the evaluation period, whereas the Real Maniqueña cultivar exhibited complete mortality within the same timeframe. In contrast, under our experimental conditions, the experiment was terminated at 8 dpi due to complete plant mortality, which coincided with the extensive production of *P. variabilis* sporangia observed across the foliage. Although no studies have directly evaluated the relationship between sporangia production and plant survival in quinoa under controlled conditions, strong associations between airborne sporangia density and disease severity have been demonstrated in other downy mildew systems. For instance, Granke et al. (2014) showed that sporangia density was the most influential factor determining disease severity of *Pseudoperonospora cubensis* in cucumber, exceeding the contribution of temperature and leaf wetness. More broadly, inoculum density and sporulation capacity are recognized as key drivers of epidemic development in other oomycete pathosystems (Fry et al., 2015; Savary et al., 2019).

Although environmental conditions were kept constant in the present study, the experimental system consisted of plastic containers containing a high density of plants. Relative humidity was increased to 90-100% for 48 h after inoculation by closing the containers with a lid. Thereafter, the lids were partially opened for the remainder of the experiment. Despite maintaining inter-plant spacing to minimize shading, this configuration may have modified the local microclimate surrounding the plants and may have contributed to the observed mortality. However, the effects of humidity and air circulation were not evaluated experimentally in this study. Nevertheless, high relative humidity, prolonged leaf wetness, and limited air circulation are well-established factors promoting sporulation and infection efficiency in downy mildews and other oomycetes (Bulovienė and Survilienė, 2006; Cohen et al., 2017; Fry et al., 2015).

The direct relationship between environmental factors, plant genotype, and *Peronospora* spp. growth remains unclear, as commonly used disease parameters such as chlorophyll content, sporulation, and plant survival represent indirect proxies of pathogen development. Previous studies have quantified *Peronospora* DNA using qPCR-based approaches; however, most focused on airborne sporangia (Klosterman et al., 2014) or soil and airborne inoculum rather than within host tissues (Van der Heyden et al., 2020). In this study, we directly quantified the relative abundance of *P. variabilis* DNA within quinoa leaf tissues, providing an estimate of pathogen accumulation during infection, as recommended for biotrophic pathogens (Schena et al., 2013; Lievens et al., 2006), and allowing comparison to other parameters.

Comparable work has been conducted in other downy mildew systems, such as *Peronospora belbahrii* infecting sweet basil; Shao and Tian (2018) reported pathogen accumulation beginning at 3 dpi, followed by a rapid increase until 5 dpi. We observed a similar trend in quinoa. During this early phase, visible symptoms and sporangia production are generally absent (Colque-Little et al., 2021b; Rollano-Peñaloza et al., 2022), suggesting that the primary source of pathogen DNA originates from internal mycelial development. In addition, results from the leaf disc assay demonstrated that *P. variabilis* DNA could be detected in infected leaves lacking visible sporulation, indicating that pathogen detection is not dependent on the presence of visible spores. Together, these observations are consistent with the biology of biotrophic oomycetes, which initially establish haustoria and expand intercellular hyphae within host tissues before external sporulation (Perfect and Green, 2001; O’Connell and Panstruga, 2006). Both our results and those of Shao and Tian (2018) indicate that these early infection stages occur predominantly within the first three days post-inoculation, although additional experiments are required to evaluate the environmental and host-related factors influencing this phase.

The identification of three consistent stages of *P. variabilis* growth in both cultivars suggests that environmental conditions may exert a strong influence during early infection, consistent with quantitative resistance models (Poland et al., 2009). The first three days post-inoculation constitute a lag phase with little *P. variabilis* DNA abundance, yet appear critical for pathogen establishment, and our results indicate that both cultivars show a similar pathogen accumulation during this phase. After a following rapid proliferation of *P. variabilis* DNA up to a peak at day 5-7, depending on experiment and coinciding with the onset of sporulation, the amount of detectable *P. variabilis* DNA again decreases to a lower level. This is likely due to different yields of extractable DNA from hyphae and spores. Such has been reported for fungi, and also oomycete spores have been reported to be recalcitrant to DNA extraction (Feng et al., 2010; Penouilh-Suzette et al., 2020). Kurmi has previously been described as tolerant to *P. variabilis* (Rollano-Peñaloza et al., 2022), whereas Pandela is commonly used for pathogen maintenance due to its susceptibility (A. PROINPA; personal communication). Although different trends in abundance of extractable pathogen DNA were observed at specific time points, the overall infection dynamics and developmental progression were comparable, making it difficult to clearly distinguish tolerant from susceptible responses based solely on qPCR or chlorophyll content.

During later stages of infection, both cultivars accumulated betalains and phenolics relative to chlorophyll levels. Kurmi showed higher phenolic to chlorophyll ratio than Pandela, indicating a cultivar-specific metabolic responses. Phenolic compounds are widely associated with defense-related processes in plant–oomycete interactions. However, despite Kurmi’s elevated phenol accumulation, pathogen DNA was not reduced. Previous reports have shown that pathogens can tolerate or detoxify these compounds (Lone et al., 2024). Changes in chlorophyll content followed by an increase in phenolic compounds has also been reported during *Bipolaris sorokiniana* infection in barley, reflecting the physiological stress imposed by pathogen colonization (Derakhshan et al., 2026). Likewise, interactions with other microorganisms, such as *Cladosporium* spp., have shown that phenolic accumulation may contribute to a priming effect, enhancing plant resistance (Derakhshan et al., 2026). Specific phenolic compounds, such as capsaicin, have been reported to inhibit hyphal development of *Phytophthora cinnamomi*, highlighting that the defensive role of phenolics depends on their composition and the particular plant–pathogen interaction (Medeira et al., 2012). The higher phenolic to chlorophyll ratio observed in Kurmi may not exclusively reflect a more effective defense response but could arise from broader metabolic reprogramming associated with infection stress, tissue damage, or differences in resource allocation between cultivars (Asghar et al., 2025; Camisón et al., 2019). Thus, while differential metabolite accumulation can distinguish resistant and susceptible genotypes, resistance or tolerance is unlikely to be explained by the accumulation of a single metabolite class alone (Camisón et al., 2019).

Building on these observations, our results provide an objective quantitative framework for evaluating the *P. variabilis* quinoa interaction, highlighting the importance of integrating physiological parameters with direct pathogen quantification. Assessing pathogen presence in asymptomatic tissues and sporulation capacity could further improve cultivar discrimination for mechanistic studies. Beyond disease monitoring, the *btub*-based qPCR assay may also be used for resistance screening in quinoa breeding, evaluating fungicide efficacy, and for studying the epidemiology of downy mildew during and between growth seasons. Although the present study focused on absolute pathogen quantification, future applications could incorporate a quinoa reference gene to normalize pathogen DNA relative to host DNA when comparisons among samples with variable DNA recovery are required. Importantly, applying this framework under field conditions will help identify the key determinants of *P. variabilis* dispersal and disease dynamics in agronomically relevant settings, ultimately supporting the development of more resilient quinoa varieties.

## 5 Conclusion

Estimation of pathogen DNA within leaf tissues allowed the identification of distinct infection stages and revealed that early pathogen establishment occurs prior to visible symptoms or sporulation. Although chlorophyll and carotenoid contents were affected during infection, these parameters alone were insufficient to clearly discriminate between tolerant and susceptible cultivars, as both Pandela and Kurmi exhibited similar infection trajectories under the tested conditions. Our results highlight the importance of integrating physiological traits with direct pathogen measurements to improve the assessment of host pathogen interactions. Future studies aimed at dissecting *P. variabilis* quinoa interactions may support the development of more robust screening strategies under both controlled and field conditions.

## Data Availability

The original contributions presented in the study are included in the article/**Supplementary Material**. Further inquiries can be directed to the corresponding author.
