# Supplementary material for: A beta-tubulin-based qPCR assay for monitoring of Peronospora variabilis and disease progression in quinoa
Source: Front Plant Sci. 2026 Sep 30;17:1872093. doi: 10.3389/fpls.2026.1872093 (PMC13661985; doi:10.3389/fpls.2026.1872093)
Supplement: Supplementary file 1 [file DataSheet1.docx]

# **Supplementary Figures**

***cox2***

**Supplementary Figure 1**. Standard curves generated using 10-fold serial dilutions of *Peronospora variabilis* genomic DNA for the (A) *cox2* and (B) *nad1* qPCR assays. The *cox2* assay showed an amplification efficiency of 77%, a slope of −4.00, and an R² of 0.9507, whereas the *nad1* assay showed an amplification efficiency of 67%, a slope of −4.49, and an R² of 0.9538.

**Supplementary Figure 2.** Linear regression between spectrophotometric chlorophyll quantification and RGB-based chlorophyll estimates in *P. variabilis*-infected quinoa cultivars Kurmi and Pandela. Chlorophyll content determined by methanolic extraction and spectrophotometric quantification was compared with chlorophyll estimates obtained using RGB measurements from a colorimeter and computational analysis of scanned leaf images. Linear regression equations and coefficients of determination (R²) are shown for each comparison. Data represent n = 193 samples.

**Supplementary Figure 3**. Pairwise relationships between photosynthetic pigments and secondary metabolites in the quinoa cultivars Kurmi and Pandela. The upper triangle corresponds to Pandela, whereas the lower triangle corresponds to Kurmi. In each panel, the variable indicated by the column is plotted on the Y axis, whereas the variable indicated by the row is plotted on the X axis. Pearson correlation coefficients (*r*) and their significance are shown in each panel. Axes limits are shared across all panels to allow direct visual comparison between cultivars. Each point represents an individual biological sample.*Asterisks indicate statistically significant Pearson correlations (*P* < 0.05; ***P* < 0.01; ****P < 0.001).*

**Supplementary Figure 4.** Thin-layer chromatography (TLC) analysis of pigment extracts from quinoa leaves. Pigments were extracted in methanol from fresh or frozen leaf material and analyzed by TLC. Methanol and methanol supplemented with MgSO₄ were included as extraction controls. Samples correspond to healthy and *Peronospora variabilis*-infected leaves of the cultivars Pandela and Kurmi collected at 1, 4, 5, 6, 7, and 8 days post-inoculation (dpi). Plates were visualized under UV light to assess pigment profiles and relative band patterns (Wagner et al., 1984) across treatments and cultivars.

**Supplementary Figure 5.** Temporal dynamics of carotenoids, phenolic compounds, and betalains during *Peronospora variabilis* infection in the quinoa cultivars Pandela and Kurmi. Data represent the mean ± standard error (n = 8 biological replicates per sampling day). Different lowercase letters (Kurmi) and uppercase letters (Pandela) indicate significant differences among sampling days within each treatment, according to one-way ANOVA followed by Tukey’s HSD test (*P* < 0.05).
