# Supplementary material for: A beta-tubulin-based qPCR assay for monitoring of Peronospora variabilis and disease progression in quinoa
Source: Front Plant Sci. 2026 Sep 30;17:1872093. doi: 10.3389/fpls.2026.1872093 (PMC13661985; doi:10.3389/fpls.2026.1872093)
Supplement: Supplementary file 2 [file Table1.docx]

# **Supplementary Tables**

**Supplementary Table 1.** Primers designed for the detection and identification of *Peronospora variabilis*.

| **Name** | **Primer Sequence (5' to 3')** | **Source** |
| --- | --- | --- |
| COX2 F | GGCAAATGGGTTTTCAAGATCC | (Hudspeth et al., 2000) |
| COX2 R | CCATGATTAATACGACAAATTTCACTAC | (Hudspeth et al., 2000) |
| btub F | ACTGGCCAAGGGACACTA | (Goker et al., 2007) |
| btub R | CACGTTAGCATTTGCTCATCA | (Goker et al., 2007) |
| nad1 F | CCACTGAACAACGTAAAGACTCTAT | (This study) |
| nad1 R | ATCGTGGTAAGATAGCCCGC | (This study) |

**Supplementary Table 2**. Temperature and relative humidity recorded in the greenhouses in Lund, Sweden, and La Paz, Bolivia. Values are presented as mean ± standard deviation (SD) and the observed range.

| **Location** | **Temperature ( ̊C), mean ± SD** | **Temperature range ( ̊C)** | **Relative humidity (%) mean ± SD** | **Relative humidity range (%)** |
| --- | --- | --- | --- | --- |
| Lund, Sweden | 19.88 **±** 1.63 | 15.20–26.05 | 49.86 **±** 32.57 | 13.1–100 |
| La Paz, Bolivia | 15.09 **± 5**.41 | 0.0–35.0 | 43.15 **±** 14.58 | 6.0–100 |

**Supplementary Table 3.** Primers designed for the quantification of *Peronospora variabilis*

| **Name** | **Primer Sequence (5' to 3')** | **Amplicon size** | **Source** |
| --- | --- | --- | --- |
| PvCOX2qF | TGGTAGTCAATGGTATTGGAGT | 198 | (This study) |
| PvCOX2qR | TGTAAAACATCCGAAGCCGT |  | (This study) |
| PvbtubqF | CTTGGTGTGTGCCGCTATGT | 118 | (This study) |
| PvbtubqR | GAAGAAGTGGAGACGGGGA |  | (This study) |
| Pvnad1qF | AGGTGCGTTAAGATCAACTGC | 144 | (This study) |
| Pvnad1qR | AGAGGAAAACAATACCAAACCGT |  | (This study) |
